# Genome-wide distribution comparative and composition analysis of the SSRs in Poaceae

**DOI:** 10.1186/s12863-015-0178-z

**Published:** 2015-02-15

**Authors:** Yi Wang, Chao Yang, Qiaojun Jin, Dongjie Zhou, Shuangshuang Wang, Yuanjie Yu, Long Yang

**Affiliations:** Key Laboratory of Crop Biology of China, Shandong Agricultural University, Taian, 271018 China; Agricultural Big-Data Research Center, Shandong Agricultural University, Taian, 271018 China; College of Plant Protection, Northwest Agriculture and Forestry University, Yangling, 712100 China

## Abstract

**Background:**

The Poaceae family is of great importance to human beings since it comprises the cereal grasses which are the main sources for human food and animal feed. With the rapid growth of genomic data from Poaceae members, comparative genomics becomes a convinent method to study genetics of diffierent species. The SSRs (Simple Sequence Repeats) are widely used markers in the studies of Poaceae for their high abundance and stability.

**Results:**

In this study, using the genomic sequences of 9 Poaceae species, we detected 11,993,943 SSR loci and developed 6,799,910 SSR primer pairs. The results show that SSRs are distributed on all the genomic elements in grass. Hexamer is the most frequent motif and AT/TA is the most frequent motif in dimer. The abundance of the SSRs has a positive linear relationship with the recombination rate. SSR sequences in the coding regions involve a higher GC content in the Poaceae than that in the other species. SSRs of 70-80 bp in length showed the highest AT/GC base ratio among all of these loci. The result shows the highest polymorphism rate belongs to the SSRs ranged from 30 bp to 40 bp. Using all the SSR primers of Japonica, nineteen universal primers were selected and located on the genome of the grass family. The information of SSR loci, the SSR primers and the tools of mining and analyzing SSR are provided in the PSSRD (Poaceae SSR Database, http://biodb.sdau.edu.cn/pssrd/).

**Conclusions:**

Our study and the PSSRD database provide a foundation for the comparative study in the Poaceae and it will accelerate the study on markers application, gene mapping and molecular breeding.

## Background

The Poaceae (Grass family) is one of the largest families of flowering plants, comprised of approximately 600 genera and 10,000 species [1]. The Poaceae plants are the world's most important food source for human beings. SSRs (Simple Sequence Repeats) are short tandem repetitive (at least 2-7) sequences of a basic unit with less than seven base pairs [2]. SSRs are widely distributed in the genomes and are widely used in biological applications such as breeding, gene location, evolution, etc. [2-4].

SSR markers are co-dominant, abundant, multi-allelic, uniformly distributed, and can be detected by simple reproducible assays [5,6]. SSR markers are more polymorphic than RFLPs (restriction fragment length polymorphism) and RAPDs (random amplified polymorphic DNAs) [3]. The high polymorphism, abundance, and convenience of SSRs make them a useful tool for genetic diversity and evolution studies [7-9].

SSR markers are ideal molecular tools for developing high density genetic maps [10]. In the past few years, an increasing number of genetic maps based on SSR markers have been developed, e.g., on rice [11], bread wheat [12] and triticale [13]. Currently, SSR markers are being used to integratee genetic maps and physical maps in plants and represent an efficient tool for breeders and geneticists to link phenotypic variations with genotypic variations [4]. Moreover, as tools for studying molecular evolution, SSR markers are used in investigating the origin, genetic diversity and dynamics of population evolution [8,14]. Furthermore, SSR markers are the commonly used markers for molecular marker assisted selection (MAS), which in turn directs molecular breeding [15,16]. The rapidly developing related studies also need more information about the SSRs.In summary, it is clear that SSR markers have significant advantages in extensive applications in plants.

The development of SSR markers requires the sequencing of the target species. Initially, SSRs were developed from expressed sequence tags (ESTs) and bacterial artificial chromosome (BAC) sequences in most plants [2]. Currently, however, more and more markers are developed based on whole genome sequences. With the development of sequencing technology, a large number of EST sequences have been determined. Besides the *Oryza sativa L. ssp. Indica* with small genome (2002) [17], several plants belonging to the grass family with huge genomes also have been sequenced, such as the *moso bamboo* (2013) [18], *Triticum aestivum* (hexaploid bread wheat) [19] and its relatives of *Triticum urartu* (wheat A-genome, 2013) [20], *Aegilops tauschii* (wheat D-genome, 2013) [21],. With the newly sequenced genomes, studies on the identification and distribution of SSRs have been performed on numbers of species [22-26]. However, genome-wide comparison of the characteristics of SSRs among different grass species have not been reported, hampering the utilization of known sequences in marker development in related species and the research on the evolution of SSRs among species.

In this study, we present the genome-wide detection of SSR sequences from nine completely sequenced grass species (*Oryza sativa L. ssp. japonica, Oryza sativa L. ssp. Indica, Zea mays, Sorghum bicolour, Brachypodium distachyon, Foxtail millet, Moso bamboo, Triticum urartu* and *Aegilops tauschii*). Specifically, the characteristics of the SSRs are described based on the abundance, density, and base ratio of different motifs and the genomic elements (EXON, INTRON, UTR and whole GENOME). Finally, we develop a freely available Poaceae SSR database which is a comprehensive platform for genetic studies and MAS.

## Results

### Statistics of the SSR loci and primers

In total, 11,993,943 SSR loci with a minimum length of 12 bp were detected from the nine grass species, and were categorized into seven types according to the unit length: monomer, dimer, trimer, tetramer, pentamer, hexamer, and heptamer (Table 1). Among these, hexamers are the most abundant (58.8%) type. There are no significant differences in the SSR abundance composition within the nine species. The SSR density of the grass family varies greatly between species, with the maximum value in rice (1611 per Mb) and the minimum value in wheat-A (771 per Mb).

**Table 1:** **Occurrence of different SSR motifs in Arabidopsis and 9 Poacead species**

**Species**	**Arabidopsis**	**Indica**	**Bamboo**	**Bread wheat**	**Maize**	**Millet**	**Japonica**	**Sorghum**	**Wheat D**	**Wheat A**	**Percentage**
**Motif**											
**Monomer**	13,678	18,689	33,840	7,987	30,918	11,288	16,456	14,292	49,289	44,524	1.94%
**Dimer**	9,389	37,542	112,470	9,430	65,250	12,420	37,447	38,202	128,784	154,577	4.89%
**Trimer**	17,509	83,231	130,017	36,997	186,749	44,075	82,572	80,378	263,191	272,427	9.66%
**Tetramer**	11,196	51,793	150,923	32,191	122,672	33,871	49,725	78,057	198,272	216,215	7.63%
**Pentamer**	3,825	16,953	45,839	7,970	38,654	9,245	17,120	16,652	43,472	45,621	1.98%
**Hexamer**	87,737	321,203	102,4250	182,645	138,5114	251,640	302,713	492,967	154,1607	1,698,582	58.82%
**Heptamer**	27,342	91,677	292,745	47,776	305,914	69,959	87,499	142,410	392,833	411,405	15.09%
**All**	170,676	621,088	1,790,084	324,996	2,135,271	432,498	593,532	862,958	2,617,448	2,843,351	100.00%
**Size(MB)**	117.01	490.02	1,996.8	262.57	2,007.04	387.96	368.27	714.44	3,256.32	3,686.4	
**No. of SSRs/Mb**	1,458.6	1,267.5	896.5	1,237.7	1,063.9	1,114.8	1,611.7	1,207.9	803.8	771.3	

A total of 6,799,910 (57%) SSR primers were designed from the identified loci (Table 2). The rest loci are not suitable for primer design either because the flanking sequences are too short or because containing high GC content. The density of SSR primers ranged from 411 per Mb (Wheat-D) to 929 per Mb (Japonica) in different species. The SSR marker density in the centromere region (469 per Mb, maize) was lower than in the chromosome arms (516 per Mb).

**Table 2:** **Statistics of the SSR primers in Poaceae**

**Species**	**Size (MB)**	**Primers**	**Primer/MB**
Indica	513.82	295,100	574.3
Bamboo	2,086.05	1,238,576	593.7
Bread wheat	275.32	244,157	886.8
Maize	2,100.87	1,066,470	507.6
Millet	406.80	308,876	759.3
Japonica	386.16	358,640	928.7
Sorghum	749.09	390,055	520.7
Wheat A	3,800.82	1,514,778	398.5
Wheat D	3,361.20	1,383,258	411.5
Average	1,520.02	755,545.56	620.1

### Characteristic of SSR loci

To analyze the frequency of different SSR motifs, SSRs are standardized first. For example, SSRs with motifs of ATG, TGA, GAT, TAC, ACT and CAT are analyzed as ATG. Among all the ten species (nine Poaceae and Arabidopsis), the most frequent motifs are in high similarity (Table 3), and most of them are abundant with AT bases except the trimer which has a higher percentage of GC bases.

**Table 3:** **The most frequent motif based on length**

**Motif length**		**Dimer**		**Trimer**		**Tetramer**		**Pentamer**		**Hexamer**		**Heptamer**	
**Species**													
*Oryza sativa L. ssp. indica*	motif	ta	ga	ggc	gag	gatc	tatt	aaaag	taaaa	tctttt	aaaata	ttttatt	aagaaaa
	number	207,422	134,238	166,702	48,150	18,631	17,477	6,159	5,168	26,483	22,309	6,381	5,432
*Oryza sativa L. ssp.japonica*	motif	ta	ga	ggc	gag	gatc	tatt	aaaag	taaaa	tctttt	aaaata	ttttatt	aagaaaa
	number	216,603	132,553	169,394	49,836	17,713	16,079	6,275	5,189	24,246	21,107	6,381	4,891
*Phyllostachys heterocycla*	motif	ta	ga	ggc	ctt	tatt	atac	taaaa	aaaag	aaaata	tctttt	ttttatt	gaggagg
	number	521,497	469,501	118,594	108,529	70,239	49,365	29,112	13,984	72,939	52,814	12,708	8,818
*Brachypodium distachyon*	motif	ga	ta	ggc	ctt	tgca	tatt	taaaa	aaaag	tctttt	aaaata	aagaaaa	ttttatt
	number	38,348	21,589	52,062	26,219	13,804	10,600	2,838	2,686	12,970	7,543	2,621	1,640
*Zea mays*	motif	ta	ga	ctt	ggc	tatt	tgca	aaaag	taaaa	tctttt	gagccc	aggaagc	ctgcttc
	number	285,957	244,863	190,564	103,855	77,682	33,766	16,133	13,116	113,450	51,720	11,119	11,111
*Foxtail millet*	motif	ga	ta	ggc	gag	agaa	gagg	aaaag	taaaa	tctttt	aaaata	ttttatt	aagaaaa
	number	90,655	45,569	63,656	35,468	11,457	10,635	3,308	1,537	15,511	10,527	3,349	3,064
*Sorghum bicolor*	motif	ta	ga	ggc	att	tatt	tgca	taaaa	aaaag	aaaata	tctttt	aggggcc	ggaccgg
	number	275,228	102,404	66,278	65,832	60,105	18,540	4,857	4,831	54,185	35,085	12,765	12,699
*Aegilops tauschii*	motif	ga	gt	ctt	gag	tatt	tgca	taaaa	aaaag	tctttt	aaaata	ttttatt	aagaaaa
	number	556,203	301,146	297,281	208,816	80,376	61,754	30,819	19,290	109,440	83,782	21,296	20,412
*Triticum urartu*	motif	ga	ta	ctt	gag	tatt	tgca	taaaa	aaaag	tctttt	aaaata	ggtggtg	ttttatt
	number	629,789	308,771	285,749	191,802	109,305	54,928	26,034	20,862	109,022	107,694	32,306	22,936
*Arabidopsis thaliana*	motif	ta	ga	ctt	aac	tatt	agaa	taaaa	gtttt	aaaata	tctttt	taaaccc	ttttatt
	number	49,195	29,738	35,167	9,971	10,479	6,064	3,514	1,623	11,160	9,407	2,845	2,498

**Figure 1:**
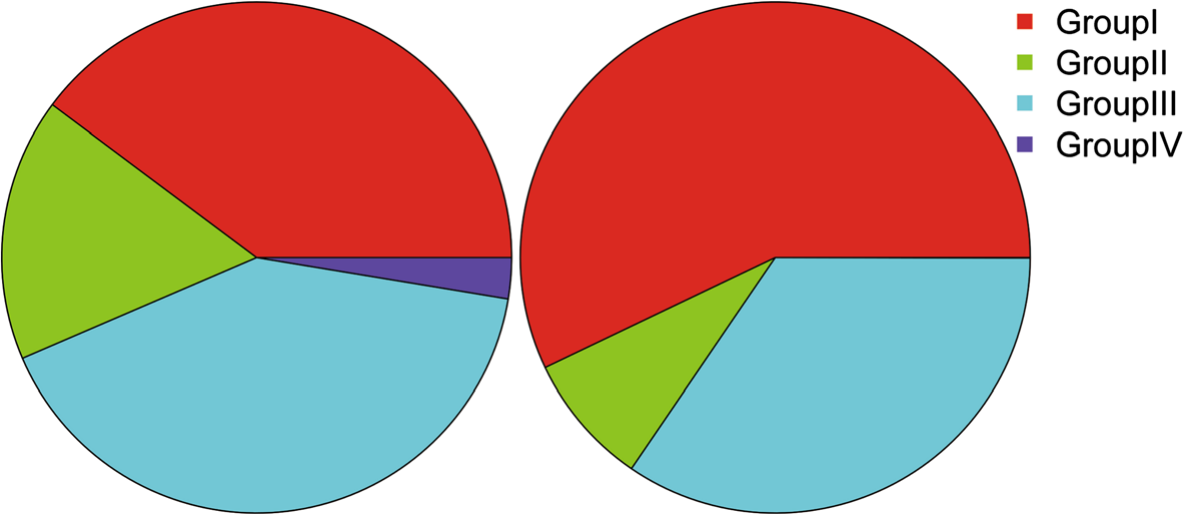
**The nucleotide composition of dimeric motif in Poaceaes and Arabidopsis.** The motifs of dimer are classified into four groups (I: AT/TA, II: AC/CA/TG/GT III: AG/GA/TC/CT IV: CG/GC). The left pie is the composition of Poaceae, the right one is *Arabidopsis thaliana.* (Better to show the value for each part).

The motifs of dimer were classified into four groups - I: AT/TA, II: AC/CA/TG/GT, III: AG/GA/TC/CT, and IV: CG/GC. The percentage of group I in Poaceae species (39.8%) is much lower than in Arabidopsis (57.1%) (Figure 1). However, the percentages of the other three groups, which contain C/G bases, are much higher in Poaceae family, especially for the group IV. The percentage of group IV is 90 times higher in the Poaceae than in Arabidopsis ( 2.6% vs 0.03%) (Figure 1). Our results clearly showed that the C/G content in the dimer type SSRs is much higher in the Poaceae (31.4%) than in Arabidopsis (21.5%).

Except in the UTRs, no significant difference of SSR coverage rate was observed in the genomic elements including exons, introns and intergenic regions (Table 4). The UTRs have a higher coverage rate than other elements (such as gene, EST, intron and CDS). For the 10 species examined, the coverage rate of the genome ranged from 1.0% (*Tritium urartu*) to 2.2% (*Japonica*). The SSRs appear on every element, and the distribution of SSRs across all the elements is relatively uniform.

**Table 4:** **Cover rate of SSRs as per genomic element**

***Species***	**Genome**	**Gene**	**CDS**	**Intron**	**UTR**
*Arabidopsis thaliana*	1.90%	1.63%	1.44%	1.68%	2.24%
*Oryza sativa L. ssp. indica*	1.67%	1.87%	1.83%	1.88%	
*Phyllostachys heterocycla*	1.26%	1.68%	1.51%	1.60%	
*Brachypodium distachyon*	1.55%	1.50%	1.49%	1.04%	1.62%
*Zea mays*	1.33%	1.61%	1.38%	0.97%	
*Setaria italica*	1.47%	1.55%	1.68%	1.44%	
*Oryza sativa L. ssp. japonica*	2.16%	1.96%	1.56%	1.37%	2.48%
*Sorghum bicolor*	1.59%	2.10%	2.02%	1.69%	3.24%
*Aegilops_tauschii*	1.06%				
*Triticum_urartu*	1.02%				

To elucidate the relationship between SSR abundance and the recombination rate, we conducted a correlation analysis between them of the maize chromosome 1 and 3. The results showed significant positive correlation (p-value <0.001) between SSR abundance and the recombination rate.

**Figure 2:**
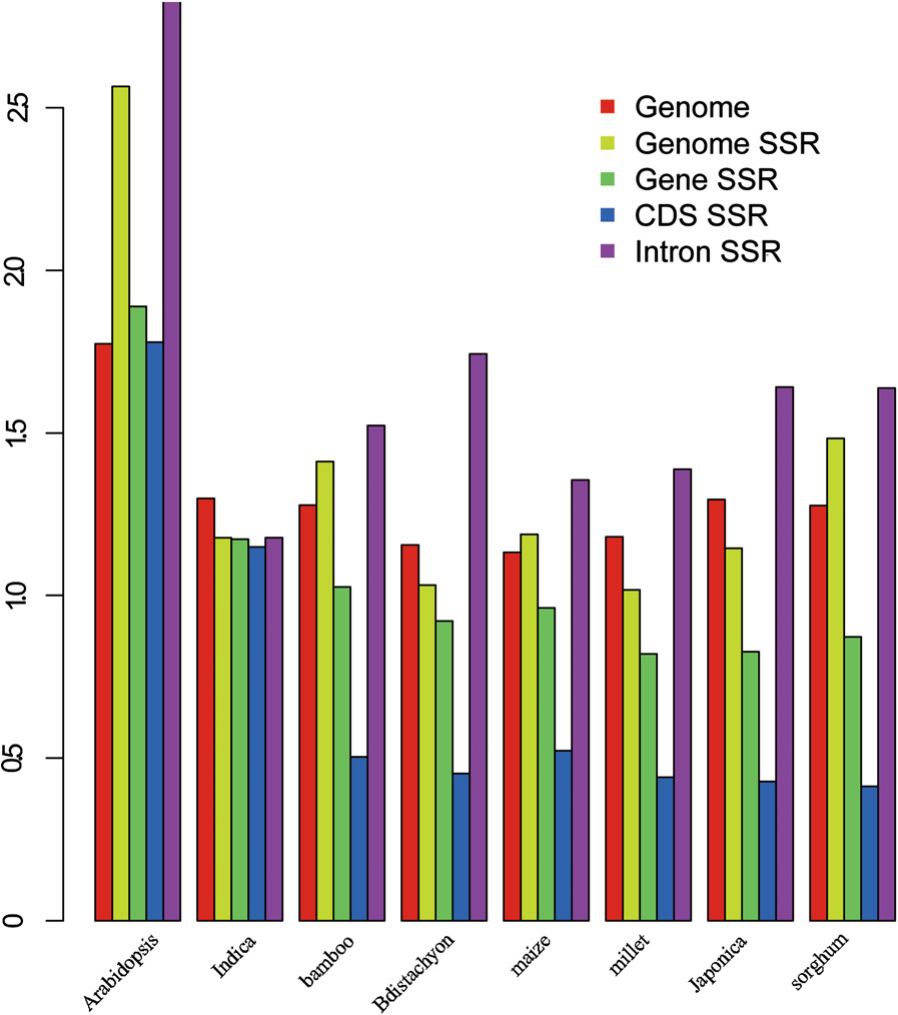
**The base ratio (AT/CG) of the genomic elements.** The genome elements include CDS SSRs, intron SSRs, gene SSRs, genome SSRs and genome.

In this study, we defined the base ratio as the number of AT/the number of CG. The base ratios of CDS (coding sequences), introns, genes and the genome in Arabidopsis are higher than those in the Poaceae (Figure 2). For all the species examined, except Indica, the base ratio of CDS is the lowest, while that of the intron elements is the highest. Interestingly, the base ratios of all the elements are the same in Indica. In summary, the base ratio of the coding sequence is lower than that of the non-coding sequence.

**Figure 3:**
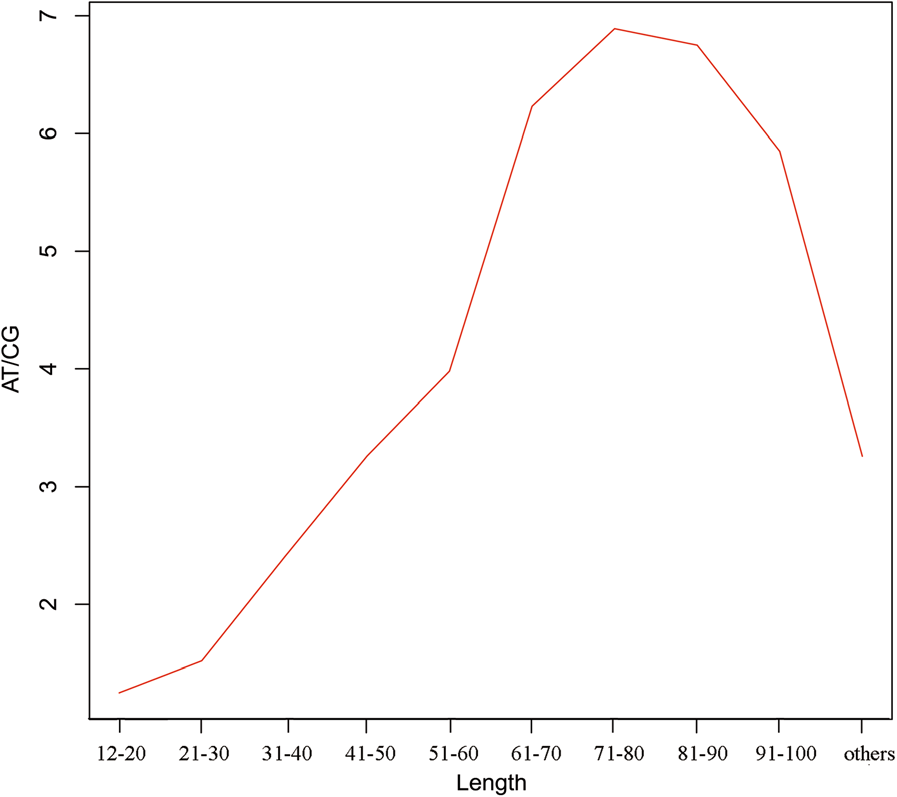
**The base ratio (AT/CG) of SSRs of different length.** The first group is SSRs of 12 bp - 20 bp, the last one is > 100 bp, and the rest are divided equally into 8 groups.

We further divided the SSR sequences into 10 groups according to their length, and analyzed the relationship between SSR length and base ratio. For the SSRs shorter than 80 bp, the length of the SSR sequences is positively correlated with the base ratio, but for the SSRs longer than 80 bp, the length is negatively correlated with the base ratio (Figure 3).

**Figure 4:**
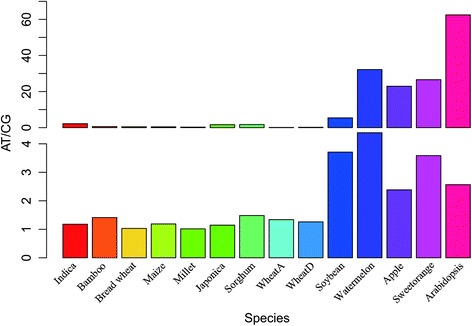
**The base ratio (AT/CG) of 14 species genome.** The figure was divided into two parts, the first part shows the base ratio of monomer and the second part showed that of the mixture from dimer to heptamer.

Using Soybean, Watermelon, Sweet Orange, Apple and Arabidopsis as an out-group, the SSR loci types were categorized into two types: the monomers and the non-monomers. The base ratio has a very wide range for the monomers (Figure 4I). In the Poaceae species, it ranges from 0.11 (Wheat A) to 2.19 (*Indica*), and the base ratio of the others are similar among the nine Poaceae species and the control group (Figure 4II). For both the monomers and the others, the base ratio is much higher in the control group than in the Poaceae species.

### Polymorphism of SSR primer

The polymorphism rate of primers varies with the length of the SSR; when the length of the SSRs is longer than 20 bp, they tend to have a higher polymorphism rate. The rate peaks at the length range from 30 to 40 bp and then oscillates but remains at a relatively stable level.

### Universal primers

Seventy-three universal primers were developed, and 19,607 e-PCR products were found on the nine genomes. Nineteen universal primers belonging to Japonica were selected randomly and their productions were anchored to the genomes of six species by MapDraw. The positions of the productions indicated that the markers have several duplications in the genomes of the grass. Additionally, some of the products were located in the mitochondrion and chloroplast. NCBI Blast also found these segments in the organelles and some unknown mRNAs, which further confirmed our findings. The sequences of some of the *in silico* amplification products are highly conserved; there are only a few SNPs (single nucleotide polymorphisms). Thus, the universal primers can be used in all the examined Poaceae species and can be used to localize genes or motifs that are linked with these sequences.

### Database content and web interface

All the information on SSR loci and SSR markers can be found in the PSSRD (Poaceae SSR Database, http://biodb.sdau.edu.cn/pssrd/index.html). PSSRD has several main pages with different functions. In the search page, SSRs can be searched by species, chromosome, motif, units, and even the start and end loci. The primers and universal primers can also be searched. An online tool for developing SSR primers is available in the tool page. The SSR loci and primers information can be downloaded in the download page.

## Discussion

### Distribution of SSRs

In the grass family, SSRs appear in all elements of the genome and are distributed throughout the chromosome. The SSRs appeared in each of the elements of the genome, indicating a wide usage of the genome. Our results are similar to those of Lawson MJ [27]. Combined with other markers, SSRs are ideal tools for studying the genome.

In our study, the abundance of SSRs is highly correlated with the recombination rate and is consistent with the studies on human SSRs [28]. There might be a direct link if recombination is mutagenic to microsatellite sequences or if the simple sequence repeats participate in the recombination to some extent; for example, if SSRs act as recombination signals or if their special composition accelerates the recombination in some way. Alternatively, recombination could exert an indirect effect by the uncoupling of natural selection at linked loci, promoting polymorphism [28]. All these data are limited by the density of the molecular linkage maps. The availability of more high density molecular linkage maps and more suitable standards for the SSRs will clarify the relationship between SSR abundance and the recombination rate.

### Implications of the base ratio

Compared with other families, the SSRs of the Poaceae species have a higher percentage of C/G bases, which means that SSRs in the grass family are more stable [29]. Stable genetic materials lead to a lower mutation rate and a longer period of evolution. It might also be the reason that the Poaceae plants contain longer chromosomes than other species. All these results are consistent with the previous studies in the monocots [29].

The higher percentage of C/G in the coding regions means a lower probability of mutation for the SSRs in the coding sequence. On the other hand, the non-coding sequences show a higher base ratio (AT/GC) and therefore have a higher probability of being mutated.

It is possible that there are excessive amounts of redundant sequences in the grass family, causing the base ratio to more closely reflect a random composition. The base ratio of the monomer varies across species.It has a very wide range, which may be due to the species’ own characteristics. And the result shows that the composition of the monomers is not random, perhaps because the number of the monomers for the coding sequences of the grass family is too small. The total number of monomers in the grass family is not less than that in the control group, but the number of monomers in the coding sequences is much less than in the other species.

The higher GC-content of the grass SSR, especially of the coding sequences, may lead to a lower expression of the grass genes compared with the controls. GC content has been proven to be correlated with recombination during meiosis [30,31]. The significant positive linear relationship of SSR abundance to recombination rate further proves our point. The high GC base ratio indicates a special evolutionary status of the Poaceae. The resultalso points to a new way of studying evolution.

### Poaceae primers

The huge number of primers designed in this database will be useful for developing genetic maps, assembling genomes, locating the genes, and breeding and will promote the study of the Poaceae.

High conservation implies a very important function and lesser evolutionary pressure to change [32]. The appearance of universal primer products in the genomes of all the examined species implies that they play essential roles in those species. Their appearance in the organelles shows the relation between the nuclear genome and the organelle genome. It also provides new evidence for the theory that the nuclear genome guides the synthesis of the organelles. The universal primer can also be used to detect the positions of some related genes, some of which might be housekeeping ones.

The polymorphism rate shows that longer SSRs may cause more polymorphisms because of the instability of long SSRs and there being many productions per primer pair in this range, which provides more opportunities for the SSRs to change. The SSRs longer than 40 bp have lower polymorphism rates, perhaps because they are too long to have enough loci per primer, and thus the chances of changes are low. This result suggests that a primer that contains a SSRs range from30 to 40 bp is more efficacious.

### Poaceae SSR database

To our knowledge, PSSRD is the first comprehensive database for SSR loci and primers in Poaceae. Data in this database is free for all academic and non-commercial users. This database will provide a huge number of markers and related information for the researchers involved in the study of Poaceae. Researchers are expected to give feedback after using the data in this database to facilitate updates and further provide more useful information to the users.

## Conclusions

Our studies of the SSRs from nine Poaceae plants show that the GC base ratio in the grass family is higher than other families. The SSRs have a high positive correlation with the recombination rate in maize. PSSRD database provides an useful resource to the comparative SSRs study in the Poaceae and will accelerate the study on markers application, the gene mapping and the molecular breeding.

## Methods

### Genome sequences source

The whole genome sequences of nine grass species were downloaded from public databases (Table 5). The genomes of *Arabidopsis thaliana*, soybean, watermelon, sweet orange, apple and PA64 (a variety of *Oryza sativa L. ssp. Indica*) were used as control groups.

**Table 5:** **Sources of genome sequences used in this study**

***Species***	**Version**	**Data source**
*Arabidopsis thaliana*	TAIR10	http://www.arabidopsis.org/
*Oryza sativa L. ssp.japonica*	Release 7.0	http://rice.plantbiology.msu.edu/
*Zea mays*	Release 5a	http://www.maizegdb.org/
*Sorghum bicolor*	Sbi1_4	http://www.phytozome.net/sorghum.php
*Oryza sativa L. ssp. indica*	full Release	http://rice.genomics.org.cn/rice/link/download.jsp
*Brachypodium distachyon*	192	ftp://ftp.jgi-psf.org/pub/compgen/phytozome/v9.0/Bdistachyon/
*Foxtail millet*	v2.0	http://foxtailmillet.genomics.org.cn/page/species/download.jsp
*Phyllostachys heterocycla*	v1.0	http://202.127.18.221/bamboo/down.php
*Triticum urartu*	v1	http://www.ncbi.nlm.nih.gov/sra/SRX047327
*Aegilops tauschii*	v1	http://www.ncbi.nlm.nih.gov/sra/SRX047430
*Oryza sativa (indica cultivar-group) PA64*	2013.8.13	ftp://public.genomics.org.cn/BGI/rice_seq/PA64s/
*Cucumis sativus*	v2.3	http://cucumber.genomics.org.cn/page/cucumber/index.jsp
*Glycine max*	Phytozome v7.0	http://www.soybase.org/dlpages/
*Sweetorange Citrus sinensis*	2012.11.29	http://citrus.hzau.edu.cn/orange/download/data.php
*Malus apple*	v1	http://www.rosaceae.org/data/download

### Genome-wide SSRs analysis

Using a Perl script, SSR loci were detected and classified into seven types according to their copy number, and all of them tandemly arranged: monomer (one copies, at least twelve repeats), dimer (two copies, at least seven repeats), trimer (three copies, at least six repeats), tetramer (four copies, at least five repeats), pentamer (five copies, at least four repeats), hexamer (six copies, at least three repeats), and heptamer (seven copies, at least two repeats).

The distribution, abundance, and base ratio (AT/CG) of the SSRs were analyzed by several Perl scripts. Based on the genetic map and the physical map [33], chromosomes 1 and 3 of maize were chosen to test the correlation between the recombination rate and the SSR abundance.

### Primers development

The SSR primers flanking 60 bp of the SSR loci were designed using eprimer3 [34]. Using the genome sequences of nine grass species, all the primers were tested and filtered by e-PCR [35]. The universal primers were selected using all the SSR primers amplified in the nine genomes, and then a polymorphism analysis was performed in silico on the genomes of Japonica, Indica and PA64. Some of the universal primers were also located on the genome using Mapdraw [36].

### Database architecture

Based on all these data, a database named PSSRD has been established. The PSSRD database (Poaceae SSR Database, http://biodb.sdau.edu.cn/pssrd/index.html) consists of some interrelated relational databases implemented in MySQL. The data handling and analysis parts of the database used pipelines written in Perl Scripts. The web interface, running on an Apache web server, was implemented in HTML. The PSSRD database was set up on a World Wide Web server allowing internet access with a web client.

## Abbreviations

SSR: Simple sequence repeats
EST: Expressed sequence tags
UTR: Untranslated region
CDS: Coding sequence
